# Overweight and glucose/lipid metabolism abnormality associated with SSRIs: a pharmacovigilance study based on the FDA adverse event reporting system

**DOI:** 10.3389/fphar.2024.1517546

**Published:** 2025-01-10

**Authors:** Jinming Cao, Zhicong Chen, Yan Wang, Yunpeng Ma, Zhen Yang, Jian Cai, Zhijun Xiao, Feng Xu

**Affiliations:** ^1^ Fengxian Hospital, Southern Medical University, Shanghai, China; ^2^ Sixth People’s Hospital South Campus, Shanghai Jiaotong University, Shanghai, China; ^3^ Fengxian Mental Health Center, Shanghai, China

**Keywords:** selective serotonin reuptake inhibitors, FAERS, overweight, ADEs, glucose/lipid metabolism disorders

## Abstract

**Background:**

In the past few decades, selective serotonin reuptake inhibitors (SSRIs) became widely used antidepressants worldwide. Therefore, the adverse reactions of patients after SSRI administration became a public and clinical concern. In this study, we conducted a pharmacovigilance study using the Adverse Event Reporting System (FAERS) database of the US Food and Drug Administration. Our main goal was to evaluate adverse events related to SSRIs, with a particular focus on abnormal weight gain and glucose/lipid metabolism disorders.

**Method:**

The adverse event data for representative SSRIs (citalopram, escitalopram, fluoxetine, fluvoxamine, paroxetine, sertraline) was extracted from the FAERS database from 2004Q1 to 2023Q4. The reporting odds ratio and proportional reporting ratio were employed to explore relevant adverse event reports (ADEs) signals. Univariate logistic regression analysis was utilized to explore factors associated with glucose/lipid metabolism abnormality following SSRIs treatment.

**Results:**

We identified 143,744 ADE reports associated with SSRIs and revealed significant abnormal signals related to weight gain and glucose/lipid metabolism in depressed patients. Variations were observed among different SSRIs medications. Specifically, citalopram was associated with abnormal weight gain (ROR: 4, 95% CI: 3.1-5.2) and hepatic steatosis (ROR: 2.8, 95% CI: 2.1-3.6); escitalopram was correlated with gestational diabetes (ROR: 9.1, 95% CI: 6.6-12.4) and cholestasis (ROR: 2.4, 95% CI: 1.75-3.38); fluoxetine was associated with obesity (ROR: 2.8, 95% CI: 2.08-3.78); fluvoxamine was linked to arteriospasm coronary (ROR: 13.87, 95% CI: 4.47-43.1); and sertraline was implicated in neonatal jaundice (ROR: 16.1, 95% CI: 12.6-20.6). Females and younger age are important risk factors for the development of associated adverse effects.

**Conclusion:**

Our study screened for adverse effects associated with abnormal glucose/lipid metabolism, such as abnormal body weight and fatty liver, in depressed patients taking selective serotonin reuptake inhibitors by utilizing FAERS database. This provides valuable insights for healthcare professionals in accepting and managing patients treated with SSRIs.

## Introduction

With the increase of life pressure, the incidence rate of depression in all age groups has increased (Thapar et al., 2022; Gundersen and Bensadon, 2023). There are various factors that can lead to depression, including school or work stress, physical illness, strained relationships with family members, and financial stress in the family (Hammen, 2018). According to statistics from the World Health Organization, depression is expected to become one of the leading causes of death worldwide by 2030 (Miret et al., 2013).

Selective serotonin reuptake inhibitors (SSRIs) are widely used antidepressants for the treatment of depression and other related mental health disorders. The research on the mechanism of action of SSRIs in treating depression is constantly evolving. Early studies have shown that the expression level of brain-derived neurotrophic factor is significantly higher in patients with major depression after fluoxetine treatment (Ghosh et al., 2015). Some researchers have proposed that SSRIs exert their effects by increasing the concentration of the neurotransmitter serotonin between neurons, thereby regulating feelings and moods (Gothert et al., 2020). A study suggests that the use of sertraline significantly decrease interleukin-6 and tumor necrosis factor α in leukocytes of patients with depression (Galecki et al., 2018). In addition, some studies have shown that SSRIs improve mood disorders such as anxiety and depression by enhancing the neurotransmission of GABA_A_ receptors (Pinna et al., 2009). Currently, citalopram, escitalopram, fluoxetine, fluvoxamine, paroxetine, and sertraline are the most used representative SSRIs for the treatment of depression. Among them, escitalopram is the s-enantiomer of citalopram, which has higher therapeutic efficacy and faster onset rate of action (Yin et al., 2023).

With the increasing use of SSRIs, public and clinical professionals are increasingly concerned about the adverse reactions of these antidepressants. In general, the common early adverse reactions encompass nausea, dizziness, headache, drowsiness, anxiety, and sexual dysfunction (Marjoribanks et al., 2013). For example, a survey indicate that SSRIs not only reduce sperm quantity and vitality in male depressive patients but also stimulate the fallopian tubes in female depressive patients, therefore negatively impacting fertility (Milosavljevic et al., 2022). In addition, SSRIs induce severe and rare adverse reactions such as gastrointestinal and intracranial hemorrhage in patients with depression (Yuet et al., 2019). However, the potential metabolic disorders after SSRI administration, such as overweight, glucose/lipid metabolism disorder, dyslipidemia and pregnancy diabetes, are still poorly understood.

The FDA Adverse Event Reporting System is maintained and managed by the US Food and Drug Administration (FDA), which supports the FDA’s post-marketing safety surveillance program for all marketed drug and therapeutic biologic products. It includes adverse event reports received by FDA from manufacturers as required by regulation along with reports directly received from consumers (such as patients, family members, lawyers) and healthcare professionals (such as doctors, pharmacists, nurses), and facilitates the monitoring of drug safety and evaluation of potential medication risks. In this study, ADE data associated with SSRIs was extracted and analyzed from FAERS from the first quarter of 2004 to the fourth quarter of 2023. We aim to uncover and evaluate signals indicative of overweight and glucose/lipid metabolism disorders, and provide insights for clinical practitioners and researchers in the field.

## Methods

### Data source

ADE reporting data submitted to the FAERS from the first quarter of 2004 to the fourth quarter of 2023 was accessed and reviewed by authors. Each quarterly dataset includes information such as drug details (DRUG), patient demographics (DEMO), adverse events (REAC), and outcomes (OUTC).

### Data processing

The generic name of the target drug was defined as citalopram, escitalopram, fluoxetine, fluvoxamine, paroxetine, and sertraline, and corresponding data are retrieved in FAERS. All quarterly DEMO, DRUG, REAC, and other report data were cleaned and organized. Subsequently, duplicate reports (retaining the latest version) were removed based on CaseID, patient name, age, country, generic names, brand names and primary suspected drug (PS). Finally, PT were identified by consulting MedDRA to further screen SSRIs-related adverse reaction reports, with emphasis on overweight, glucose/lipid metabolism disorder.

### Signal mining and collation

This study utilized the Reporting Odds Ratio (ROR) and Proportional Reporting Ratio (PRR) methods for signal analysis, as delineated in Tables 1, 2. An adverse event signal was deemed significant if it met the following criteria: ADE reports >3, ROR >2, PRR >2, lower limit of the 95% confidence interval (CI) for ROR >1, lower limit of the 95% CI for PRR >1, and χ2 >4. Subsequently, manual screening was conducted to identify signals associated with overweight and glucose/lipid metabolism disorders. All data processing and signal mining calculations were performed using R software (version 4.2.2). P value less than 0.05 was considered statistically significant.

**TABLE 1:** Disproportionality analysis.

Drugs	Number of targeted ADE reports	Number of other ADE reports	Total
Target drugs	a	b	a + b
Other drugs	c	d	c + d
Total	a + c	b + d	a + b + c + d

**TABLE 2:** Formulas and threshold values of ROR and PRR.

	Formulas	Threshold
ROR	ad/bc	*exp(ln(ROR)±1.96* $\sqrt{1/a+1/b+1/c+1/d}$ *)*
PRR	a (a + b)/c (c + d)	*exp(ln(PRR) ± 1.96* $\sqrt{1/a+1/a+b+1/c+1/c+d}$ *)*

## Results

### ADE reports overview

**FIGURE 1:**
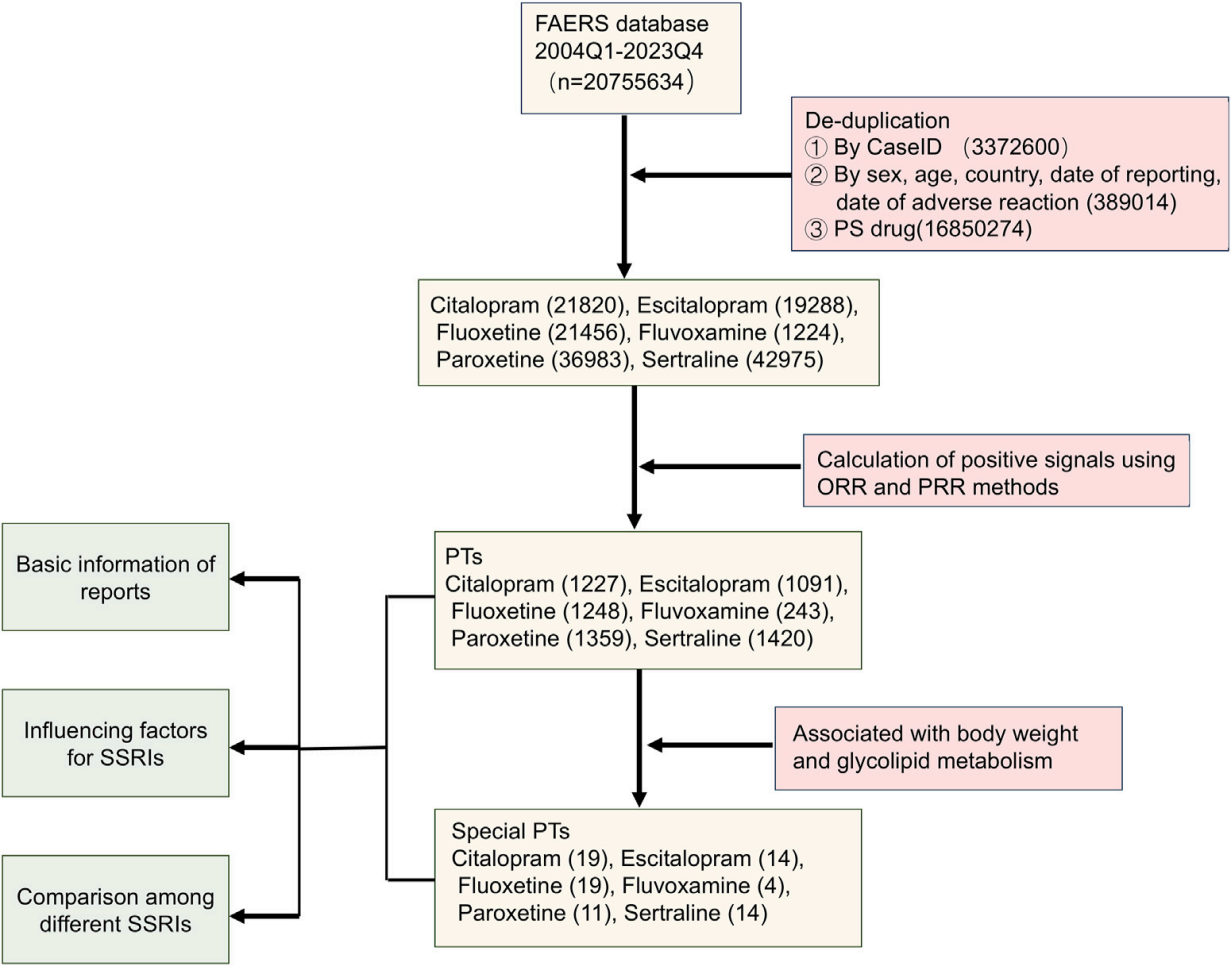
The flowchart for screening signals of overweight and glucose/lipid metabolism disorders associated with SSRIs.

Figure 1 illustrated the comprehensive data processing workflow of this study. A total of 20,755,634 ADE reports were extracted from FAERS. Following deduplication and data cleaning, a total of 143,746 reports were identified with SSRIs as the primary suspected (PS): 21,820 reports for citalopram, 19,288 for escitalopram, 21,456 for fluoxetine, 1,224 for fluvoxamine, 36,983 for paroxetine, and 42,975 for sertraline. After screening based on criteria such as ROR and PRR, a total of 6,588 PTs related to SSRIs were identified. Specifically, there were 1,227 PTs associated with citalopram, 1,091 with escitalopram, 1,248 with fluoxetine, 243 with fluvoxamine, 1,359 with paroxetine, and 1,420 with sertraline. Then, after reviewing the literature, 81 PTs related to weight and glucose/lipid metabolism were identified. Finally, we conducted a basic analysis of reports of PTs associated with SSRIs.

**FIGURE 2:**
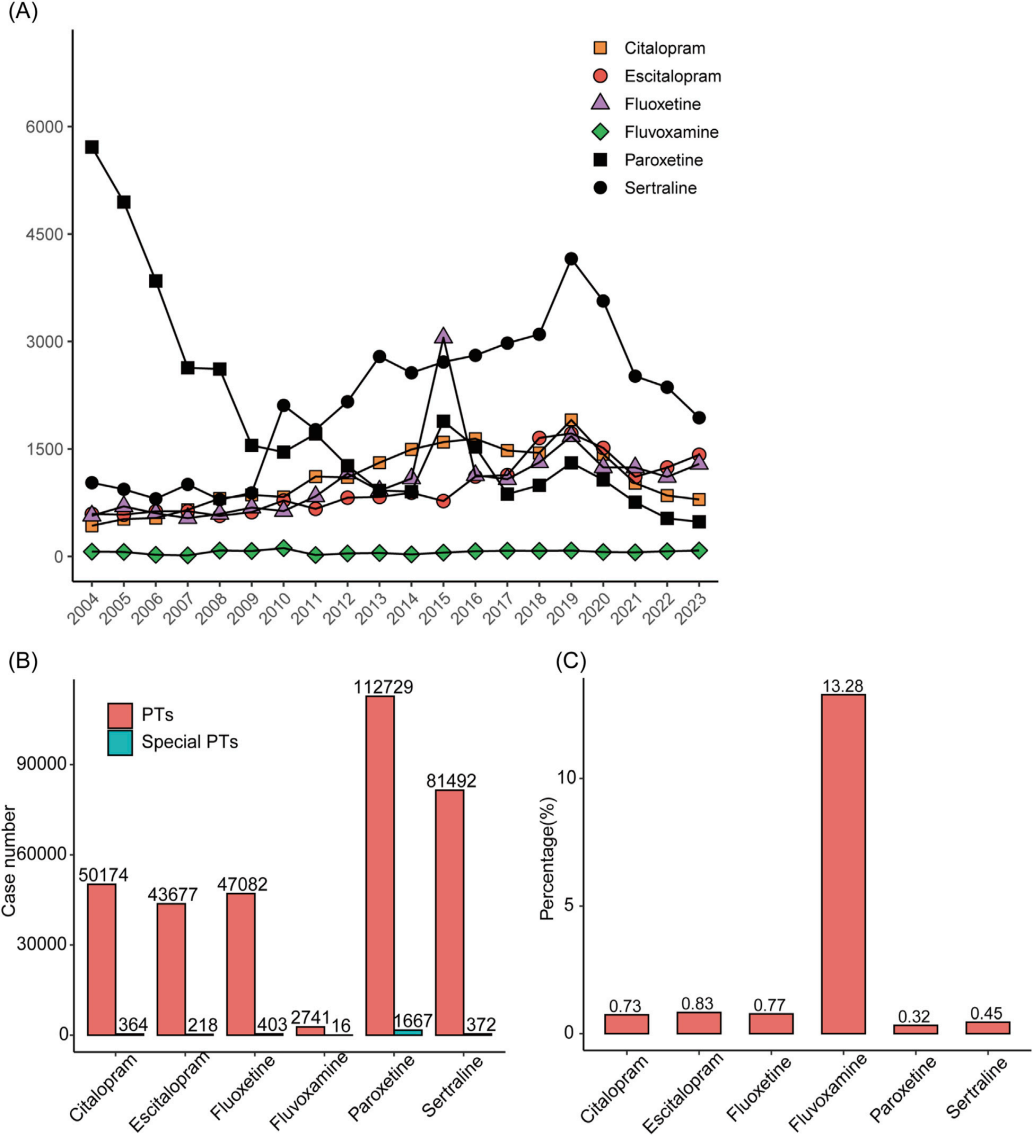
Statistics of reported diseases associated with abnormalities of glycolipid metabolism caused by treatment with SSRIs with and without SSRIs in FAERS between 2004 and 2023. **(A)** Annual ADE reports on SSRIs. **(B)** Total number of cases of adverse reactions after taking SSRIs and number of cases of adverse reactions related to disorders of glucolipid metabolism. **(C)** The proportions of reports with and without diseases associated with abnormalities of glycolipid metabolism for different SSRIs.

We visualized the number of reports for the 6 SSRIs by year (Figure 2A). The results showed a relatively smooth change in the number of ADE reports with escitalopram, citalopram, and fluvoxamine as the main suspected drugs. The number of ADE reports related to paroxetine showed an almost yearly decreasing trend. However, the number of ADE reports for sertraline increased and then decreased, with large fluctuations. We then counted the number of reports of all ADE to SSRIs and the number of reports of ADE associated with disorders of glucolipid metabolism separately (Figure 2B). Paroxetine had the highest number of adverse reaction reports, followed by sertraline. Fluvoxamine had the lowest number of adverse reactions. In addition, we calculated the percentage of the number of adverse reactions reports related to disorders of glucolipid metabolism (Figure 2C), with fluvoxamine having the highest percentage (13.28%) and paroxetine having the lowest (0.32%).

In all relevant ADE reports, the proportion of women was higher than that of men, indicating that women might be more prone to adverse reactions compared to men after taking SSRIs. The age distribution of reports was divided into four stages. Compared to the other two age groups, the number of ADE reports was higher in the 20-40 and 40-60 age groups (Table 3). Medical doctors (MD), occupational therapists (OT), and clinical nurses (CN) were the primary reporters. The majority of reports come from the United States (US), United Kingdom (GB), Denmark (DK), France (FR), Japan (JP), and Germany (DE) (Table 4).

**TABLE 3:** Characteristics of cases occurred SSRIs-related ADE.

Characteristics		Citalopram (N = 21,820)	Escitalopram (N = 19,288)	Fluoxetine (N = 21,455)	Fluvoxamine (N = 1224)	Paroxetine (N = 36,982)	Sertraline (N = 42,975)
Gender [n (%)]	Female	11,699 (53.62)	11,020 (57.13)	12,261 (57.15)	599 (48.94)	21,275 (57.53)	24,282 (56.5)
	Male	7,034 (32.24)	5,959 (30.89)	5,691 (26.53)	512 (41.83)	11,997 (32.44)	12,792 (29.77)
	Unknown	3,087 (14.15)	2,307 (11.96)	3,503 (16.33)	113 (9.23)	3,710 (10.03)	5,901 (13.73)
Age [n (%)]	<20	1800 (8.25)	1707 (8.85)	3,257 (15.18)	185 (15.11)	3,364 (9.1)	5,269 (12.26)
	20–40	4,517 (20.7)	3,603 (18.68)	4,157 (19.38)	316 (25.82)	5,498 (14.87)	8,740 (20.34)
	40–60	4,872 (22.33)	3,833 (19.87)	4,325 (20.16)	244 (19.93)	5,832 (15.77)	7,758 (18.05)
	>60	4,317 (19.78)	3,638 (18.86)	2,690 (12.54)	211 (17.24)	4,699 (12.71)	7,364 (17.14)
	Unknown	6,314 (28.94)	6,507 (33.74)	7,026 (32.75)	268 (21.9)	17,589 (47.56)	13,844 (32.21)

**TABLE 4:** The occupational distribution and national source distribution of SSRI related ADE reporters.

	Citalopram (N = 21,820)		Escitalopram (N = 19,288)		Fluoxetine (N = 21,455)		Fluvoxamine (N = 1224)		Paroxetine (N = 36,982)		Sertraline (N = 42,975)	
Reporter [n (%)]	OT	7,209 (33.04)	CN	5,339 (27.68)	CN	7,269 (33.88)	MD	326 (26.63)	CN	21,295 (57.58)	CN	17,062 (39.7)
	CN	4,429 (20.3)	OT	5,249 (27.21)	OT	3,608 (16.82)	CN	238 (19.44)	MD	5,530 (14.95)	MD	9,139 (21.27)
	MD	4,347 (19.92)	MD	4,550 (23.59)	MD	5,724 (26.86)	OT	180 (14.71)	OT	3,339 (9.03)	OT	7,015 (16.32)
	Others	5,835 (26.74)	Others	4,150 (21.52)	Others	4,854 (22.62)	Others	480 (39.22)	Others	6,818 (18.44)	Others	9,759 (22.94)
Reporting country [n (%)]	US	5,980 (27.41)	US	7,293 (37.81)	US	10,475 (48.82)	US	393 (32.11)	US	16,432 (44.43)	US	19,254 (44.8)
	GB	5,046 (23.13)	DK	3,327 (17.25)	GB	3,534 (16.47)	JP	211 (17.24)	GB	3,856 (10.43)	GB	8,744 (20.35)
	DK	2,434 (11.15)	FR	1432 (7.42)	FR	1296 (6.04)	DE	124 (10.13)	JP	2077 (5.62)	DE	1730 (4.03)
	Others	8,360 (38.31)	Others	7,236 (37.52)	Others	6,150 (28.66)	Others	496 (40.52)	Others	14,617 (39.52)	Others	13,247 (30.82)

### ADE signal detection

**FIGURE 3:**
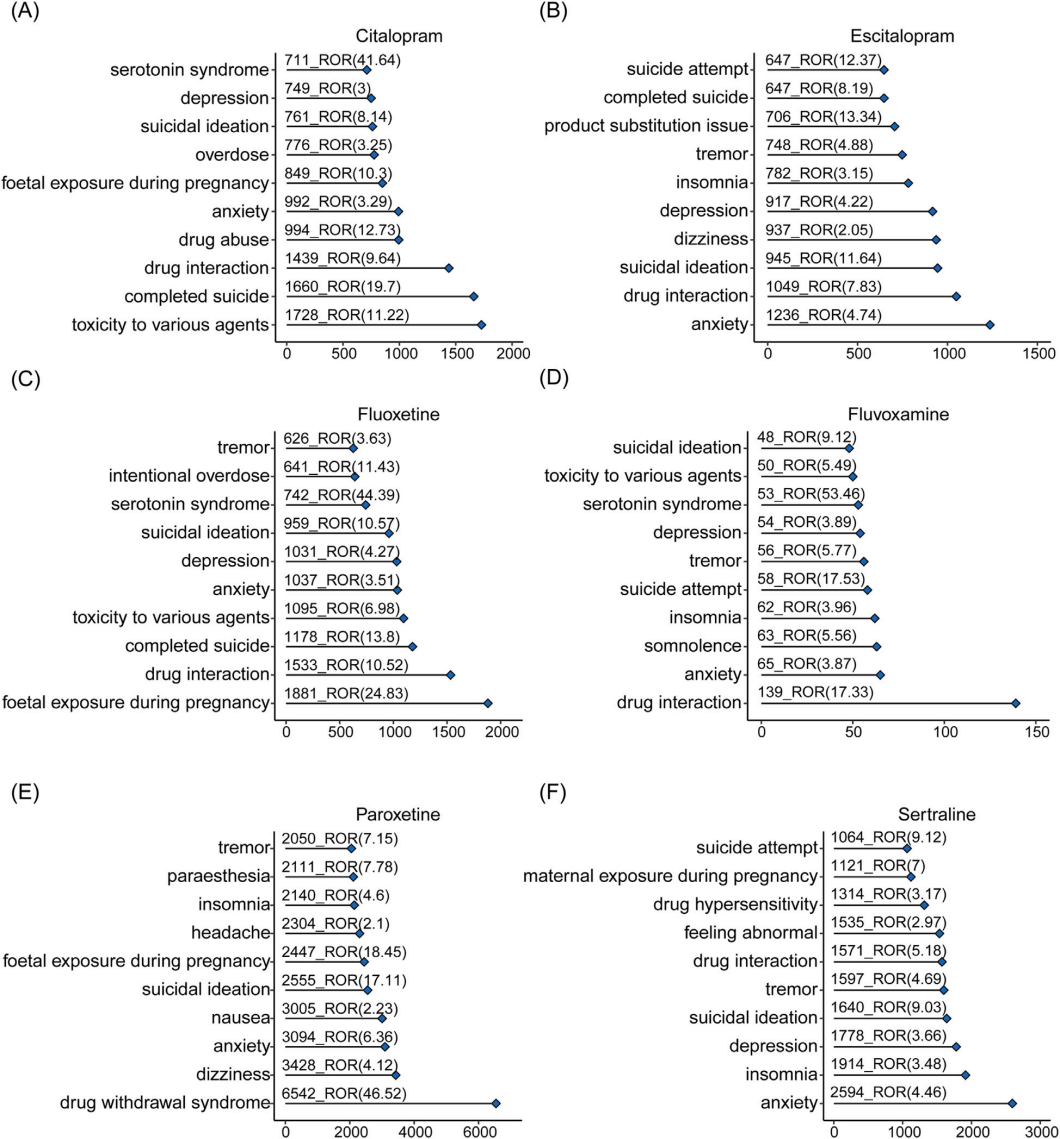
The overall signals of disorders associated with SSRIs. Adverse reactions related to citalopram **(A)**, escitalopram **(B)**, fluoxetine **(C)**, fluvoxamine **(D)**, paroxetine **(E)**, sertraline **(F)** and occurring in the top 10 numbers.

**FIGURE 4:**
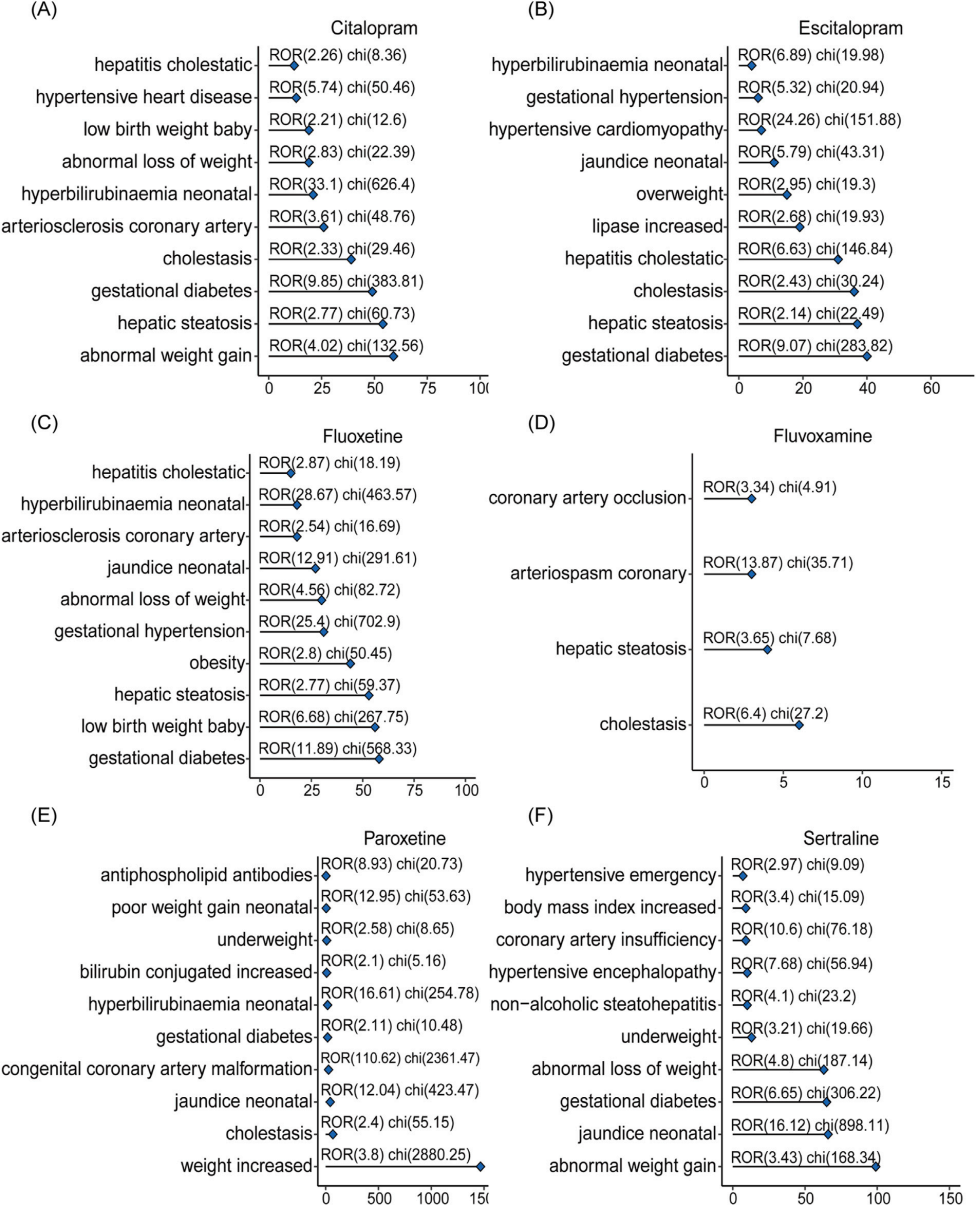
The signals of overweight and glucose/lipid metabolism disorders associated with SSRIs. Adverse reactions related to citalopram **(A)**, escitalopram **(B)**, fluoxetine **(C)**, fluvoxamine **(D)**, paroxetine **(E)**, sertraline **(F)** caused by abnormalities in glucolipid metabolism.

The top-ranking PTs in ADE signal intensity for citalopram, escitalopram, fluoxetine, fluvoxamine, paroxetine, and sertraline were shown in Figures 3A–F. The frequency of PT reports related to overweight and glucose/lipid metabolism disorders for SSRIs were 364, 218, 403, 16, 1667, and 372, respectively, as delineated in Figures 4A–F. Specifically, with the exception of fluvoxamine, the remaining five SSRIs had a higher incidence of gestational diabetes in female patients. Citalopram, paroxetine and sertraline tend to cause abnormal weight gain in patients. In addition, hepatobiliary disorders such as “hepatic steatosis” and “cholestasis” caused by abnormalities in glucose and lipid metabolism were more common with citalopram, escitalopram and fluoxetine. For more details, please refer to Supplementary Tables S1–S6 (Sheet 1 and Special PTS, which respectively include all PTs related to citalopram, PTs related to glucose/lipid metabolism abnormality).

**FIGURE 5:**
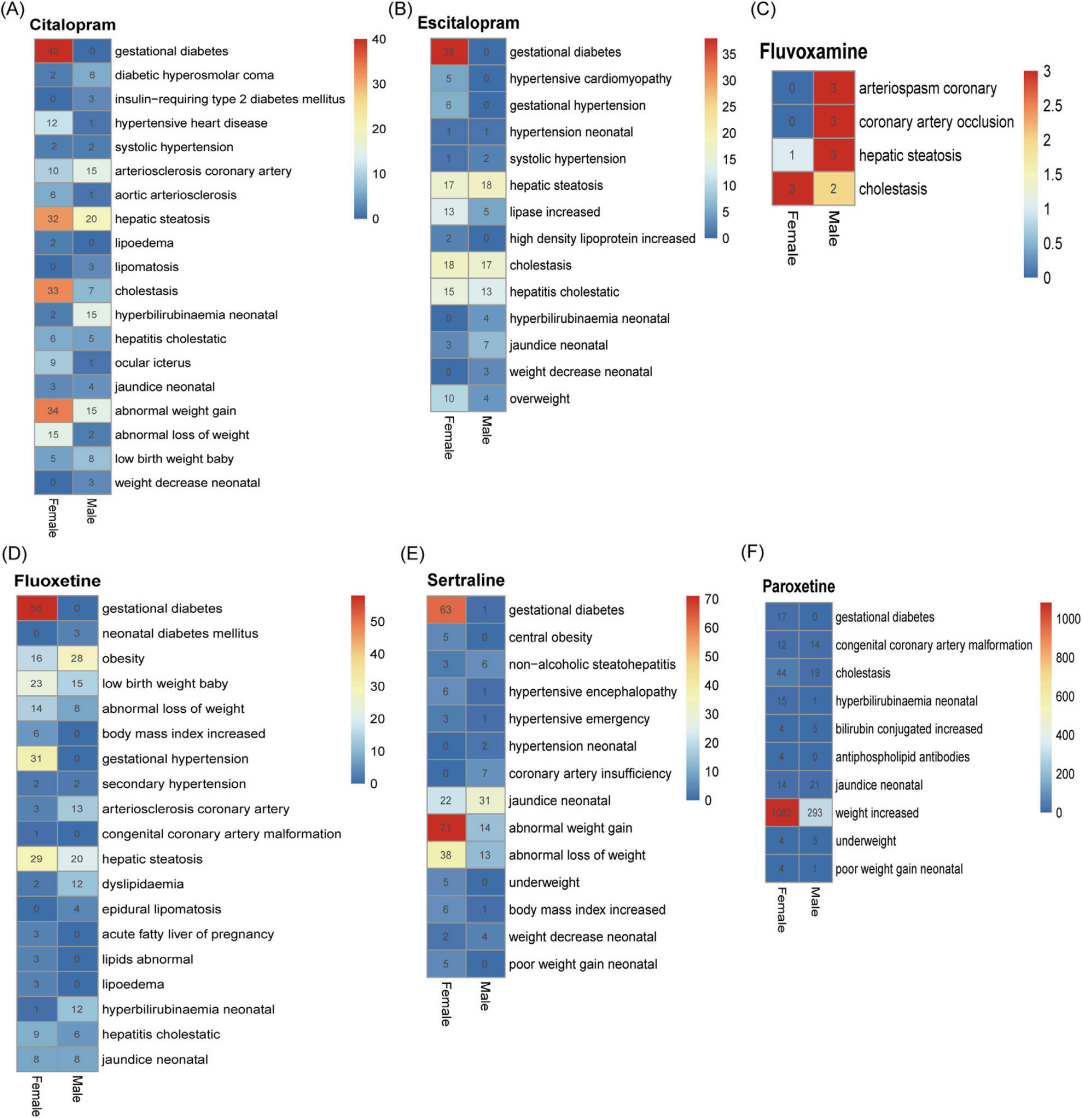
Differences in adverse reactions triggered by abnormalities in glucolipid metabolism in men and women after taking SSRIs. **(A)** Citalopram. **(B)** Escitalopram. **(C)** Fluvoxamine. **(D)** Fluoxetine. **(E)** Paroxetine. **(F)** Sertraline. The numerical values in the heatmap represent the number of specific PTs occurrences in males and females, respectively.

The number of adverse reactions caused by abnormalities in glucose/lipid metabolism was analyzed by gender (Figure 5). The results showed that, with the exception of “gestational diabetes”, “abnormal increase” and “hepatic steatosis” were more likely to occur in female depressed patients, and coronary atherosclerosis was more likely to occur in male depressed patients.

### Influencing factors for SSRIs-related glucose/lipid metabolism disorders

**FIGURE 6:**
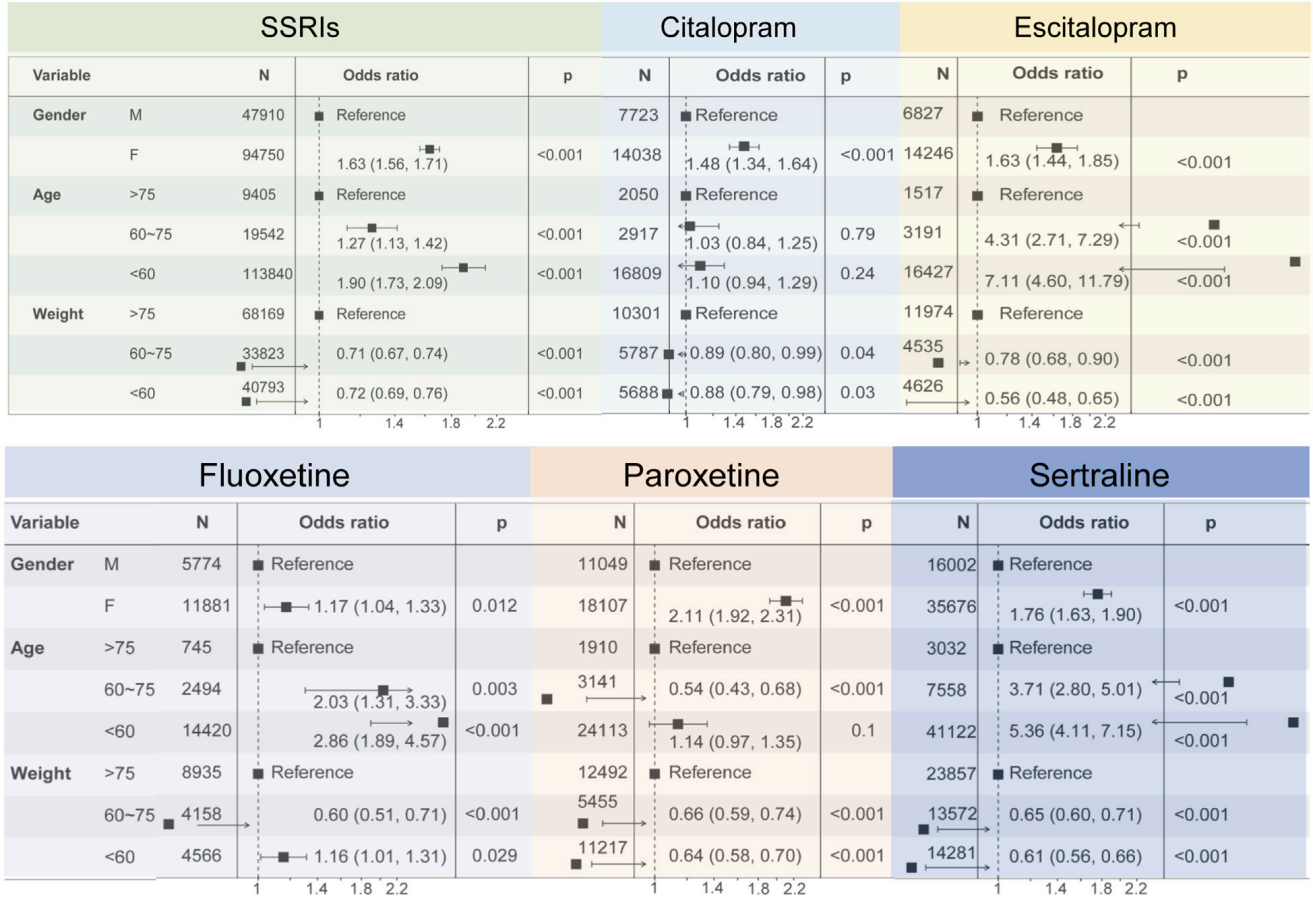
Forest plots showing the results of univariate logistic regression analyses regarding demographic factors affecting diseases caused by SSRIs-associated disorders of glucolipid metabolism.

We further investigated demographic factors that may influence the occurrence of disorders associated with glucose/lipid metabolism disorders using one-way logistic regression analyses based on case reports in which SSRIs were the primary suspected drug (Figure 6). Of these reports, only a total of 142,787 case reports of PTs associated with SSRIs all contained information on age, sex, and body weight, as detailed in Supplementary Table S7. Among all cases of SSRIs, female patients had a higher risk of glucose/lipid metabolism disorders-related disorders compared to males (OR = 1.63 [1.56, 1.71], P < 0.001). Patients aged 60–75 years were 1.27 times more likely to develop associated disorders compared to patients aged 75 years or older (OR = 1.27 [1.13, 1.42], P < 0.001), and patients younger than 60 years were 1.9 times more likely to develop associated disorders (OR = 1.90 [1.73, 2.09], P < 0.001). Patients weighing 60–75 kg and less than 60 kg had a reduced probability of developing the associated disease compared with patients weighing more than 75 kg. Subsequently, we analyzed each of the six SSRIs individually and showed that, consistent with the results of the overall analysis, both sex and age of the patients increased the probability of developing diseases associated with glucose/lipid metabolism disorders. We were unable to perform one-way logistic regression analyses for case reports in which fluvoxamine was the primary suspect drug because of the lack of information and the insufficient number of reports associated with the occurrence of diseases related to glucose/lipid metabolism disorders.

## Discussion

### Number of ADE reports

In this work, we conducted a pharmacovigilance study using the Adverse Event Reporting System (FAERS) database of the US Food and Drug Administration, focusing on representative SSRIs (citalopram, escitalopram, fluoxetine, fluvoxamine, paroxetine, and sertraline) as well as signals of overweight and glucose-lipid metabolism disorders. Among representative SSRIs, citalopram and escitalopram were the main antidepressants for the treatment of moderate to severe depression, with the latter exhibiting superior efficacy (Cipriani et al., 2012; Yin et al., 2023). In addition, fluoxetine was indicated for patients with moderate depression but might have unsatisfied efficacy in severe cases (Kishi et al., 2023). These three antidepressants were the most widely used in clinical practice. Our work confirmed that there are more reports in the real world about ADE related to overweight and glucose/lipid metabolism disorders associated with citalopram, escitalopram, and fluoxetine. Clinical application of fluvoxamine was late, therefore there were significantly fewer ADE reports for fluvoxamine compared to the other five SSRIs. Relatively speaking, both paroxetine and sertraline were effective in the treatment of moderate to severe depression, with significant efficacy and good tolerability, and fewer reports of adverse events (Sanchez et al., 2014).

### Abnormal bodyweight change, overweight or obesity

In this work, we noticed that SSRIs have various abnormal signals related to body weight. Patients taking citalopram and escitalopram might experience abnormal bodyweight change, overweight, even obesity. However, fluoxetine displayed a contrary pattern, with ADE reports of weight loss. The ADE reports of paroxetine and sertraline showed that after taking these two antidepressants, some patients gained weight while others lost weight. Currently, the mechanisms underlying abnormal bodyweight change, overweight or obesity associated with SSRIs medication remained unclear and warranted extensive research exploration. It might have been related to uncontrolled eating during depressive episodes or improvement in depressive symptoms (McCuen-Wurst et al., 2018; Yu et al., 2023). As to weight loss, a research found that three serotonin receptors (5-HT_1B_, 5-HT_2C,_ and 5-HT_6_) might have been associated with abnormal weight loss in patients (Halford et al., 2005). Activation of 5-HT_1B_ and 5-HT_2C_ receptors could have led to reduced appetite, with the latter especially believed to induce satiety through endogenous hypothalamic serotonin subtypes (Lam et al., 2010). In addition, a meta-analysis integrating data from 15 studies involving 1,977,446 subjects found that prenatal depression patients who took SSRIs medication experienced newborn weight loss (Zhao et al., 2018). Although this study did not eliminate the influence of other confounding factors, its findings deserve careful attention. However, another study analyzed 238 pregnant women (71 severe depression patients with SSRIs exposed, 36 severe depression patients with SSRIs unexposed, 131 non-severe depression patients with SSRIs unexposed) and concluded that SSRIs medication was not associated with neonatal weight loss (Wisner et al., 2013). In summary, based on our findings from FAERS data analysis and other work, large-sample real world investigation was further needed to determine whether there was an association between body overweight and SSRIs drugs.

### Gestational diabetes

Pregnant women were highly susceptible to depression and were often prescribed by physician with antidepressants for treatment. Our results indicated a risk signal for gestational diabetes with all SSRIs except fluvoxamine. A study found that SSRIs not only activate the apoptosis of pancreatic β-cell, but also promote insulin resistance and thereby triggering diabetes (Isaac et al., 2018). Instead, a review summarized that SSRIs do not increase the risk of gestational diabetes (Lebin and Novick, 2022). Therefore, it should be vigilant to the risk of gestational diabetes for pregnant women with depression if they took SSRIs during pregnancy.

### Gender differences

In this paper, our findings showed that gender was a risk factor for the occurrence of adverse reactions related to abnormalities of glycolipid metabolism after taking SSRIs. Women were more likely to experience adverse effects compared to men with depression. It was suggested that the high prevalence of depression in women may have been related to the interaction of a number of factors, including genetic, physiological, psychological, and social factors (Blanco et al., 2015; Sramek et al., 2016). These factors may have simultaneously affected women’s sensitivity and tolerance to SSRI drugs, thereby increasing their risk of adverse effects. For different SSRIs drugs, the mechanism of inducing adverse effects may have varied. For example, it was shown that fluoxetine may have induced a series of adverse reactions by affecting various neurotransmitter systems such as the 5-hydroxytryptamine system, the norepinephrine system, and the dopamine system (Kobayashi et al., 2012; Edinoff et al., 2021). As for sertraline, it may have caused adverse reactions in patients by affecting molecular targets such as 5-HT receptors and NMDA receptors (Izumi et al., 2024). Therefore, further clarification of the specific mechanisms of different SSRIs in the treatment of depression was beneficial for healthcare professionals to more accurately judge and manage the occurrence of adverse reactions.

### Lipid metabolism

Lipid metabolism was crucial for life activity in maintaining energy balance and hormone synthesis. Disorders in lipid metabolism could lead to various diseases such as obesity, hyperlipidemia, and cardiovascular diseases (Banzi et al., 2015). From our analysis on ADE reports, we found that SSRIs medications were highly prone to cause lipid metabolism disorders, such as hepatic steatosis, arteriosclerosis, cholestasis, and neonatal jaundice. However, there were not many reports on the risk signals of neonatal jaundice related to SSRIs. Although the impact of SSRIs during pregnancy on maternal and infant health was very important, the relevant research was not sufficient up to now.

## Limitations

Although the FDA Adverse Event Reporting System serves as a critical repository for regulatory oversight, pharmaceutical surveillance, and public health monitoring, its utility is tempered by inherent limitations. FAERS relies on voluntary reporting, which introduces potential biases and underreporting. In addition, the database may suffer from data duplication and inaccuracies. Moreover, FAERS lacks comprehensive patient data, including detailed medical histories and comorbid conditions. It is imperative to recognize and address these limitations for clinicians and researchers to derive meaningful insights and make informed decisions based on FAERS data.

## Conclusion

We used real-world data from the FAERS database to screen for adverse reactions associated with abnormalities in glucose/lipid metabolism. Through the proportional imbalance method, we identified PTs that were highly associated with SSRIs. In addition, there were differences in adverse reactions associated with abnormalities in glucose/lipid metabolism elicited by different SSRIs. Specifically, the adverse reactions associated with abnormalities of glycolipid metabolism associated with paroxetine were relatively low in percentage, while the absolute lowest number was fluvoxamine. Therefore, healthcare professionals should consider factors such as inter-drug variability, patient age, and gender when selecting drugs for SSRIs in order to more effectively manage the occurrence of adverse reactions.

## Data Availability

The original contributions presented in the study are included in the article/Supplementary Material, further inquiries can be directed to the corresponding authors.
